# Dataset on assessment of heavy metals contamination in multi-environmental samples from Patna, India

**DOI:** 10.1016/j.dib.2019.104079

**Published:** 2019-05-31

**Authors:** Kumari Sonu, Ishwar Chandra Yadav, Amrendra Kumar, Ningombam Linthoingambi Devi

**Affiliations:** a Department of Environmental Science, Central University of South Bihar, SH-7, Gaya-Panchanpur Road District-Gaya 824236, Bihar, India; b Department of International Environmental and Agricultural Science (IEAS), Tokyo University of Agriculture and Technology (TUAT) 3-5-8, Saiwaicho, Fuchu, Tokyo 1838509, Japan

**Keywords:** Heavy metals, Soil, Contamination, Risk

## Abstract

Accumulation of heavy metals in vegetables adversely affects the well-being of human health. In this study, we investigated the heavy metals (Hg, Zn, Cu, Pb and Mn) contamination in different environmental samples collected from five major sites (Gaighat, Paijawa, Danapur, Ranipur and Marchi) of Patna. In all the samples concentration of manganese (Mn) was found to be higher in soil samples. The concentration of heavy metals in soil samples were in the order Mn > Zn > Cu > Pb > Hg in water sample; Mn > Zn > Pb > Cu > Hg, and in vegetables Mn > Zn > Cu > Pb > Hg. In all sites, majority of heavy metal were within the permissible limits except the Zn and Pb. The Zn and Pb contents in vegetables and soil were measured above the permissible limit recommended by WHO/FAO (2007) and Indian standard. The bioconcentration factors (BCFs) for the heavy metal transfer from soils to vegetables are analysed and were ranked in the order of Hg > Pb > Zn > Cu > Mn. The estimated daily intake of metals suggested low health risk despite higher metal content in soil/vegetables. The metal pollution index (MPI) analysis showed high MPI for spinach (15.6) followed by red spinach (14.0) whereas beans (8.6) showed lower metal pollution index.

**Table:** Specifications table

Subject area	Environmental sciences
More specific subject area	Geochemistry and heavy metal pollution
Type of data	Table, figure and graph
How data was acquired	Atomic absorption spectroscopy (AAS), Thermoscientific, Ice 3000 Series
Data format	Raw and analyzed
Experimental factors	Water samples were collected using polyethylene bottles immersed in an open drain that was being used for irrigation purposes. 1mL of concentrated HNO_3_ was added in the bottle filled with water to avoid microbial utilization of heavy metals immediately after filling. Soil samples were collected in triplicate from the field receiving wastewater regularly for irrigation at the depth of 0–20 cm layer which is known to be plough layer. Vegetable samples were collected from the same field simultaneously. Care was taken to get samples of the same varieties and age group from different selected sites and delicately analysis of samples were done via AAS, Thermoscientific, Ice 3000 Series.
Experimental features	Standard methods were followed for the purpose of collecting samples, taking it to laboratory, preserving water, preparing samples (cutting, drying and grinding) and analysis.
Data source location	Patna city, Bihar, India.
Data accessibility	Detailed data are conveyed in this article

**Table:** 

**Value of the data** • This data will be important for the assessment of contamination level of heavy metals by human activities. It will be more informative for researchers for their further research. • These data's will be important for estimating health risk posed by heavy metal contamination in soil and vegetables. • Present data may be useful for policy makers, government official, stakeholder to forumulate health risk managment plan.

## 1 Data

**Fig. 1:**
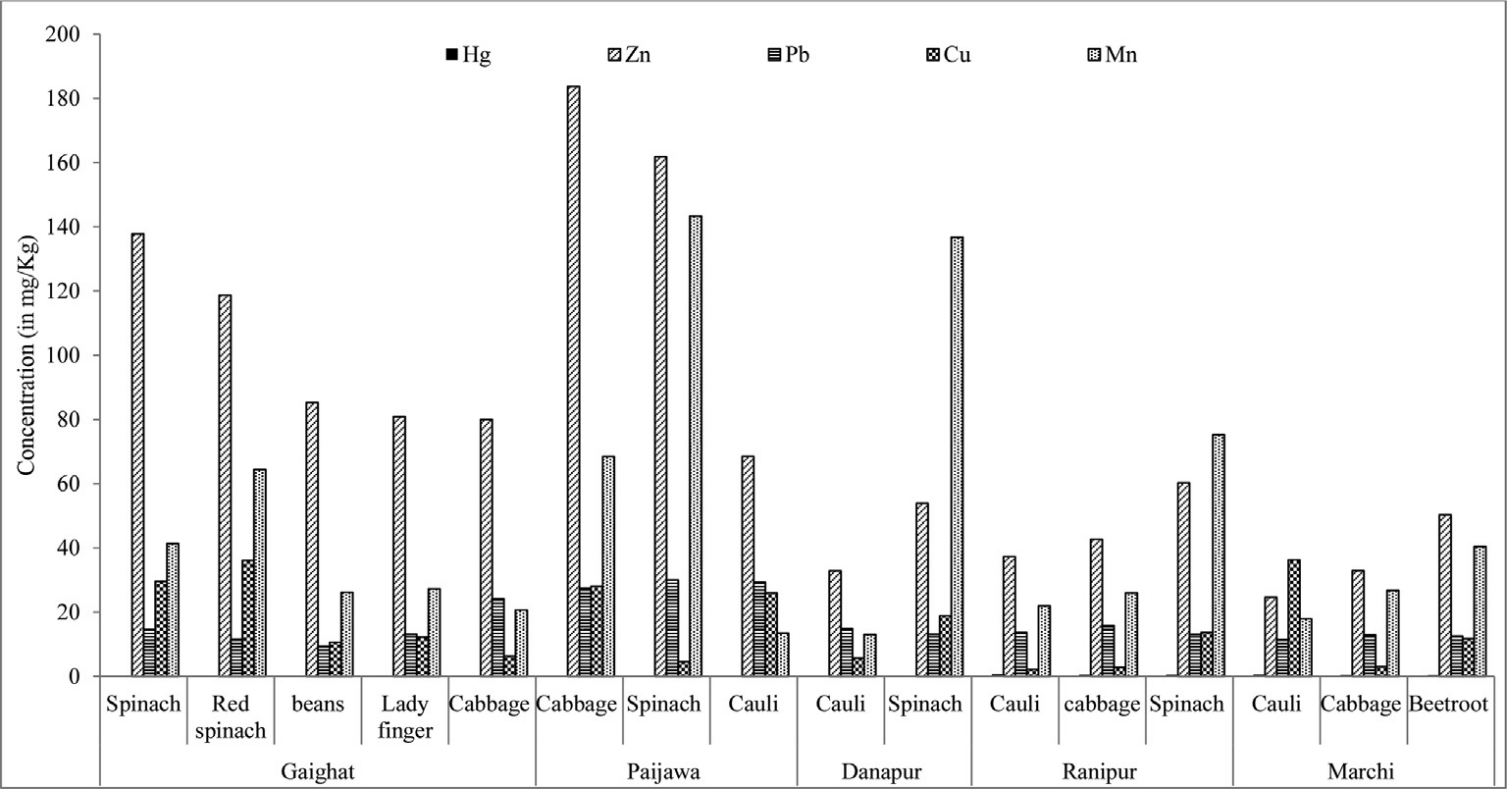
Concentration of heavy metals in vegetable samples collected from wastewater irrigated sites of Patna city.

Different concentration levels of heavy metals in water, soil and vegetables from sampling sites Gaighat, Paijawa, Danapur, Ranipur and Marchi are given in Table 1 and Table 2. Occurrence of heavy metals in the wastewater was in an order of Mn > Zn > Cu > Pb > Hg. Among all concentration of manganese in soil was found to be higher followed by Zn, Cu, Pb and Hg respectively. Concentrations of heavy metal were below the safe limits prescribed by WHO/FAO (2007) [1] and Indian standards [2]. However, heavy metal concentrations in vegetables are in the order of Zn > Mn > Cu > Pb > Hg respectively (Fig. 1).

**Table 1:** Concentrations of Heavy metals in Water and Soil samples across the sampling sites of Patna city.

Heavy metals		Water (mg/L)	Permissible limit			Permissible limit	
			WHO/FAO (2007)	Indian Standard (2000)	Soil (mg/Kg)	WHO/FAO	Indian Standard (2000)
Hg	Range	0.001–0.008			0.18–0.923		
	Mean	0.006			0.445		
Zn	Range	0–0.223	2.0	5.0	76.11–347.46		300–600
	Mean	0.065			136.952		
Pb	Range	0.058–0.066	5.0	0.10	14.53–57.42		250–500
	Mean	0.061			24.454		
Cu	Range	0–0.018	0.20	0.05	31.22–119.61		135–270
	Mean	0.003			49.604		
Mn	Range	0.124–0.338	0.2	0.1	298.92–642.48		
	Mean	0.239			417.73

**Table 2:** Heavy metal concentration (in milligrams per kilogram dry weight) in vegetable samples grown in wastewater-irrigated agricultural field.

Sampling site	Vegetables	Heavy Metal concentration in mg/Kg				
		Hg	Zn	Pb	Cu	Mn
Gaighat	Spinach	0.240	137.84	14.61	29.65	41.37
	Red Spinach	0.169	118.72	11.66	36.16	64.48
	Bean	0.211	85.26	9.42	10.64	26.20
	Lady finger	0.191	80.88	13.17	12.27	27.31
	Cabbage	0.154	80.02	24.2	6.30	20.72
Paijawa	Spinach	0.189	161.82	30.08	4.56	143.36
	Cabbage	0.171	183.72	27.44	28.10	68.48
	Cauliflower	0.145	68.52	29.34	26.02	13.52
Danapur	Spinach	0.269	53.91	13.15	18.87	136.75
	Cauliflower	0.186	32.88	14.90	5.73	13.09
Ranipur	Spinach	0.542	60.32	13.11	13.67	75.28
	Cabbage	0.541	42.73	15.84	2.83	26.03
	Cauliflower	0.705	37.32	13.68	2.17	21.96
Marchi	Cabbage	0.455	32.94	12.95	3.11	26.81
	Cauliflower	0.592	24.66	11.57	36.3	17.96
	Beetroot	0.393	50.31	12.61	11.78	40.43
WHO/FAO (2007)			60	5	40	
IS (2000)			50	2.5	30

### 1.1 Bioconcentration factor (BCF)

The bioconcentration factors (BCFs) estimated for the heavy metal transfer from soils to vegetables are shown in Table 3. The BCF values of the heavy metals such as Hg, Zn, Pb, Cu, and Mn were found to be in the ranges of 0.2–0.9, 0.2 to 1.5, 0.2 to 1.9, 0.1 to 0.5, and 0.1 to 0.2, respectively. The trend in the BCF for heavy metals in the sampling sites was in the ranking order of Hg > Pb > Zn > Cu > Mn. The food chain (soil-plant-human) is mainly known as one of the major pathways for exposure of human to soil contaminants.

**Table 3:** Bio-concentration of different metals in vegetables irrigated with waste water of Patna city.

Heavy metals	Gaighat	Paijawa	Danapur	Ranipur	Marchi
Hg	0.209	0.569	1.261	1.928	0.921
Zn	0.289	1.534	0.481	0.577	0.472
Pb	0.254	1.919	0.836	0.769	0.851
Cu	0.158	0.582	0.393	0.195	0.545
Mn	0.12	0.216	0.173	0.111	0.044

Soil-to-plant transfer is one of the key processes of human exposure to toxic heavy metals through the food chain. When BCF <1 or BAF = 1, it denotes that the plant only absorbs the heavy metal but does not accumulate when BCF >1, and this indicates that plant accumulates the heavy metals. BAF values of Hg, Zn and Pb were found to be greater than one which means that they are not only absorbing the heavy metals but also they are accumulating the heavy metals concentration which may cause health problems for human whereas BAF values of Cu and Mn were found to be less than one in the vegetable samples which indicates that plants only absorb the heavy metals not accumulate them [3].

### 1.2 Daily intake of metals (DIM)

**Fig. 2:**
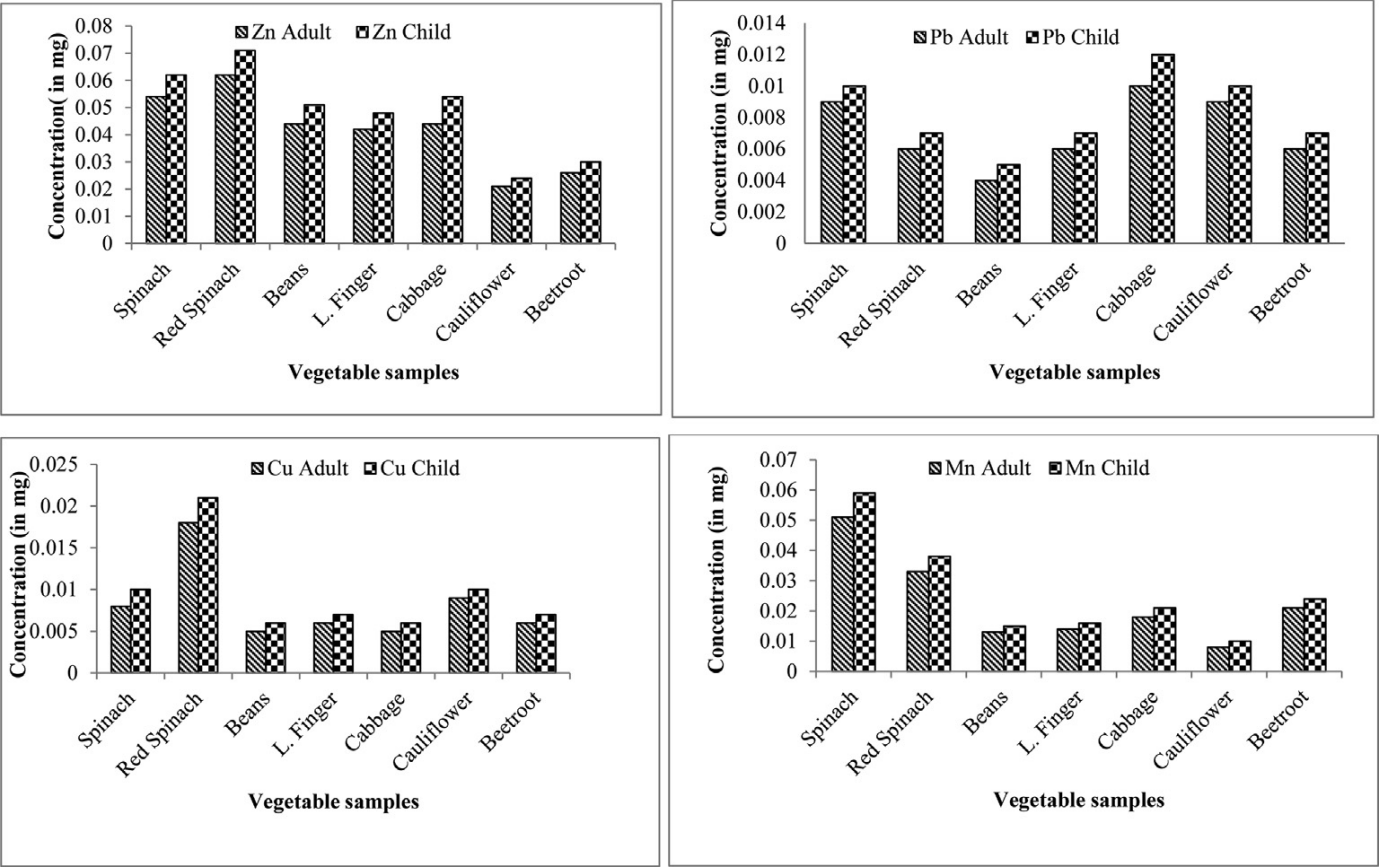
Comparison of heavy metals intake by adult and children through vegetables irrigated with waste water of Patna city.

Assessing the exposure level and tracing the path of contaminants to the observed organisms are of great value as a result to observe the underlying health risks (see Fig. 2). There may be several pathways but food chain is proved to be the important pathway of human exposure heavy metals. DIM in adults as well as children were calculated using the average amount of vegetable consumed by them on daily routine (Table 4). Fig. 2 shows the DIM in adults and children from different vegetables. DIM values for heavy metals were found to be almost negligible for Hg but were found to be highest in case of Zn, Mn, Pb and Cu from the consumption of spinach, red spinach, cabbage and beans respectively, for both adults and children, grown in wastewater-irrigated soils. DIM suggest that the consumption of vegetables grown in wastewater contaminated soils is high, but it is nearly free of risks, as the dietary intake limits of Cu, Zn and Mn in adults can range from 1.2 to 3.0 mg, 5.0–22.0 mg and 2.0–20.0 mg, respectively [4].

**Table 4:** Daily intakes of metals (in mg) for individual heavy metal in different vegetables grown in wastewater - irrigated agricultural area.

Plants	Hg		Zn		Pb		Cu		Mn	
	Adult	Child	Adult	Child	Adult	Child	Adult	Child	Adult	Child
Spinach	0.000	0.000	0.054	0.062	0.009	0.010	0.008	0.010	0.051	0.059
Red Spinach	0.000	0.000	0.062	0.071	0.006	0.007	0.018	0.021	0.033	0.038
Beans	0.000	0.000	0.044	0.051	0.004	0.005	0.005	0.006	0.013	0.015
L. Finger	0.000	0.000	0.042	0.048	0.006	0.007	0.006	0.007	0.014	0.016
Cabbage	0.000	0.000	0.044	0.054	0.010	0.012	0.005	0.006	0.018	0.021
Cauliflower	0.000	0.000	0.021	0.024	0.009	0.010	0.009	0.010	0.008	0.010
Beetroot	0.000	0.000	0.026	0.030	0.006	0.007	0.006	0.007	0.021	0.024

### 1.3 Metal pollution index (MPI)

Calculation of Metal pollution index (MPI) is effective method for monitoring of metal pollution in wastewater irrigated areas [5]. Among all collected different vegetable samples, spinach (15.659) showed highest value of MPI followed by red spinach (14.039), and beans (8.607) showed lower metal pollution index (Table 5). Higher MPI value of spinach, red spinach and cabbage recommend that leafy vegetables are potential in causing more human health risk due to the increased accumulation of heavy metals in the vegetables.

**Table 5:** Metal pollution index in different foodstuffs from wastewater irrigated site.

Samples	Metal Pollution Index
Spinach	15.659
Red spinach	14.039
Beans	8.607
Lady finger	9.262
Cabbage	11.505
Cauliflower	9.664
Beetroot	10.349

## 2 Experimental design, materials and methods

### 2.1 Study area

**Fig. 3:**
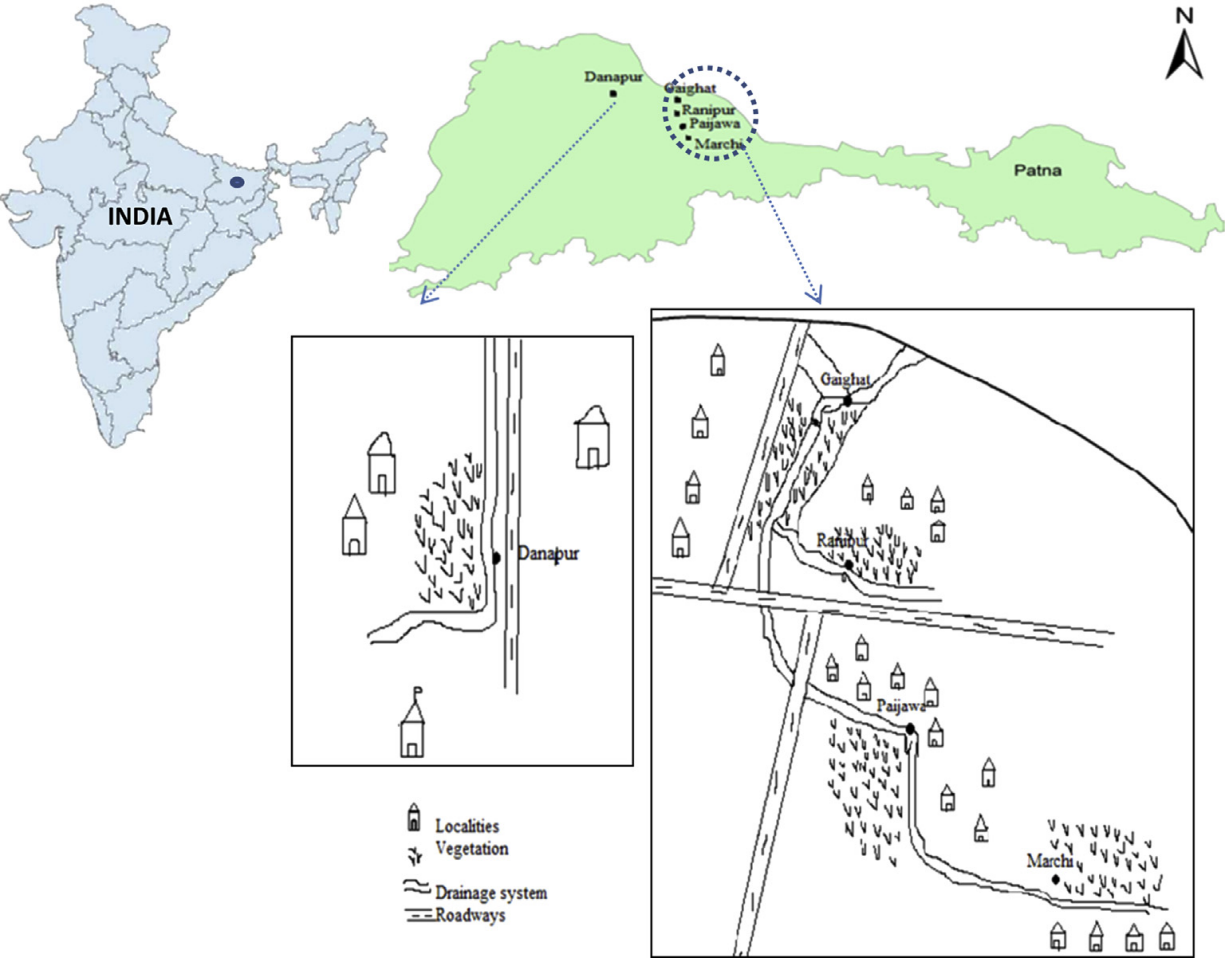
Detailed of Sampling sites of Patna city.

The multi-environmental samples were collected from Patna city (25.6°N 85.1°E), Bihar, located in the southern bank of the river Ganga in Eastern India. The total area of Patna is 136 km^2^ (53 sq mi). The municipal area constitutes 99 km^2^ (38 sq mi), while the suburban area constitutes 36 km^2^ (14 sq mi). It has an average elevation of 53 m (174 ft). It had an estimated population of 1.68 million in 2011, making it the 19th largest city in India. With over 2 million people, its urban agglomeration is the 18th largest in India. Surveillance was done in urban and semi-urban areas of Patna city to find out those sites were industrial wastewater, municipal and domestic sewage is used for irrigation purpose. Five sampling sites were identified were wastewater is direct source of irrigation including Danapur, Gaighat, Ranipur, Paijawa and Marchi were vegetables are grown using wastewater (Fig. 3).

### 2.2 Sampling of soil, water and vegetables

Samples of water, soil and vegetables were collected from five different sites namely Danapur, Gaighat, Ranipur, Paijawa and Marchi located in Patna city. Samples were collected in the morning between 6:00 a.m.–10:00 a.m. from the irrigation sites from December to February in 2016–2017. However, the samples of water were collected immersed in an open drain that was being used for irrigation purposes. 1mL of concentrated HNO_3_ was added in the bottle filled with water to avoid microbial utilization of heavy metals immediately after filling. Soil samples were collected in triplicate from the field receiving wastewater regularly for irrigation at the depth of 0–20 cm layer which is known to be plough layer. Vegetable samples were collected from the same field simultaneously. Care was taken to get samples of the same varieties and age group from different selected sites. Samples of seven different kinds of vegetables; leafy vegetables included Spinach (*Spinacia oleracea*), Red spinach (*Amaranthus dubius)* and Cabbage (*Brassica oleracea L. Var. Capatuta*). Inflorescence vegetable included Cauliflower (*Brassica oleracea L. Var. botrytis*), Fruit vegetables included Lady's Finger (*Abelmoschus esculentus L*.), Beetroot (*Beta vulgaris*), and Beans (*Phaseolus vulgaris)* were taken from the same experimental sites where waters and soils samples were taken.

### 2.3 Preparation of sample

Samples of vegetables were then washed using clean water and then air dried till constant weight was achieved. The samples were then crushed separately through a steel grinder and the crushed material was then passed through 2-mm sieve. The sieved samples were kept at ambient temperature before analysis.

For the analysis of heavy metals, 100 ml of water samples were digested after adding 10 ml. of concentrated nitric acid (HNO_3_) at 80 °C. The heating was continued, until the solution appeared transparent. After cooling the digested samples were then diluted up to 100 ml with distilled water [6].

For Soil and vegetables samples, 0.5 g of dried samples was digested with 10mL of HNO_3_ at 250 °C in tefflon bomb on hot plate until a transparent solution was obtained. The solution was then filtered through Whattman No. 42 filter paper and the solution was finally diluted to 50mL with distilled water [7].

### 2.4 Analysis of sample

#### 2.4.1 Determination of heavy metals

The prepared samples were then analysed by using Atomic Absorption Spectrophotometer (AAS, Thermoscientific, Ice 3000 Series). The AAS value of blank solution was taken for each heavy metal.

### 2.5 Data analysis

#### 2.5.1 Bioconcentration factor (BCF)

It refers to the ratio of concentration of metal in plant parts to the metal concentration in the soil.

In order to assess the concentration of metals from soil to vegetables, the BCF values of metals were calculated as follows:

BCF = C_vegetables_/C_soil_

Where, C_vegetable_ and C_soil_ represent the concentration of heavy metals in vegetables and soils, respectively [2], [10].

#### 2.5.2 Daily intake of metals (DIM)

The daily intake of metals (DIM) was calculated using the following equation:

DIM = M × K × I*/*W

Where,

M = Concentration of heavy metals in plants (mg kg^−1^),

K = Conversion factor,

I = Daily intake of vegetables,

W = Average body weight,

At first, weights of the fresh vegetables were converted into dry weight by using the value of conversion factor 0.085, as described previously [8]. The average body weights of the adult and child were considered to be 55.9 and 32.7 kg, respectively, while the average daily intakes of vegetable for adults and children's were considered to be 0.345 and 0.232 kg/person/day, respectively [9].

#### 2.5.3 Metal pollution index (MPI)

To analyse the overall concentration of heavy metal in all the vegetable samples collected from the different wastewater irrigated site, metal pollution index (MPI) was used. This index was obtained by calculating the geometrical mean of concentrations of all the metals in the vegetables [10].

MPI (mg/Kg) = (Cf_1_ × Cf_2_ × …….. × Cf_n_)^1/n^

Where, Cf_n_ = concentration of metal n in the sample.
